# Comparing CT and MR Properties of Artificial Thrombi According to Their Composition

**DOI:** 10.3390/diagnostics13101802

**Published:** 2023-05-19

**Authors:** Rebeka Viltužnik, Aleš Kaučič, Aleš Blinc, Jernej Vidmar, Igor Serša

**Affiliations:** 1 Jožef Stefan Institute, 1000 Ljubljana, Slovenia; rebeka.viltuznik@ijs.si (R.V.); jernej.vidmar@mf.uni-lj.si (J.V.); 2 Institute of Physiology, Faculty of Medicine, University of Ljubljana, 1000 Ljubljana, Slovenia; 3 Clinical Institute of Radiology, University Medical Centre Ljubljana, 1000 Ljubljana, Slovenia; ales.kaucic@gmail.com; 4 Department of Vascular Diseases, University Medical Centre Ljubljana, 1000 Ljubljana, Slovenia; ales.blinc@kclj.si; 5 Division of Internal Medicine, Faculty of Medicine, University of Ljubljana, 1000 Ljubljana, Slovenia; 6 Institute of Anatomy, Faculty of Medicine, University of Ljubljana, 1000 Ljubljana, Slovenia

**Keywords:** artificial blood clots, thrombosis, Hounsfield units, NMR relaxation, apparent diffusion coefficient, structure and composition

## Abstract

This study aims to determine whether and to what extent the structure and composition of thrombi can be assessed using NMR and CT measurements. Seven different thrombus models, namely, six RBC thrombi with hematocrit levels (HTs) of 0%, 20%, 40%, 60%, 80% and 100% and one platelet thrombus model, were analyzed using proton NMR at 100 MHz and 400 MHz, with measurements of *T*_1_ and *T*_2_ NMR relaxation times and measurements of the apparent diffusion coefficient (ADC). In addition, the thrombus models were CT-scanned in a dual-energy mode (80 kV and 140 kV) and in a single-energy mode (80 kV) to measure their CT numbers. The results confirmed that RBC thrombi can be distinguished from platelet thrombi by using ADC and CT number measurements in all three settings, while they cannot be distinguished by using *T*_1_ and *T*_2_ measurements. All measured parameters allowed for the differentiation of RBC thrombi according to their HT values, but the best sensitivity to HT was obtained with ADC and single-energy CT measurements. The importance of this study also lies in the potential application of its results for the characterization of actual thrombi in vivo.

## 1. Introduction

Thrombosis is the localized clotting of blood creating partial or complete blockage within venous or arterial blood vessels, limiting the natural flow of blood. Depending on the site of formation or clot embolization, this is associated with cardiovascular dysfunction, ischemic cerebrovascular stroke or acute pulmonary embolism. Thrombosis is a result of a hemostasis imbalance causing the over-coagulation of blood [1,2,3]. Hemodynamic conditions also play an important role in the formation of thrombi and strongly influence their structural and mechanical properties, as shown by numerous mathematical modeling studies [4,5]. The structure of thrombi was proven to affect the success of thrombosis treatment, with thrombi with a higher percentage of red blood cells (RBCs) being less prone to dissolution with thrombolytic treatment and more prone to fragmentation during mechanical thrombectomy than thrombi with a lower percentage of RBCs [6,7,8,9,10,11]. Pure plasma thrombi (0% RBC) are suggested to be more difficult to extract during mechanical thrombectomy than thrombi containing RBCs, because they tend to stick to the vessel wall more [6,12]. While CT is the most commonly used medical imaging modality in the diagnosis of acute thrombosis, due to its speed and availability, MRI has shown greater potential in defining thrombus structure and, consequently, in determining the most appropriate treatment option and providing a better possible treatment outcome [13,14,15]. CT also has some limitations when it comes to the imaging of small acute strokes, or strokes in the posterior fossa [16]. However, with new upcoming technological and image processing advances, MRI is closing the gap of protocol time frame compared to CT, with some institutions already using MRI as the modality of choice for acute stroke imaging—the average magnetic resonance (MR) protocol time is 13 min (10–16 min), while the average time for the CT protocol is 9 min (7–12 min) [17]. Nevertheless, imaging is usually only used for differential diagnosis, the approximate location and size of the occlusion and access to the occlusion in the case of mechanical thrombectomy, without special attention to the characterization of the occlusion composition, which could be the key to determining the optimal course of treatment [16]. The aim of this study is to investigate on model thrombi how their composition, especially the level of hematocrit (HT) or platelet inclusions, affects their MR and CT properties. While some previous studies have already reported the examination of thrombus structure using MR and CT, they were performed using one of these two modalities separately on different sets of samples. To the best of our knowledge, this is the first study to combine these two modalities on the same set of samples measured at different time points.

## 2. Materials and Methods

**Figure 1:**
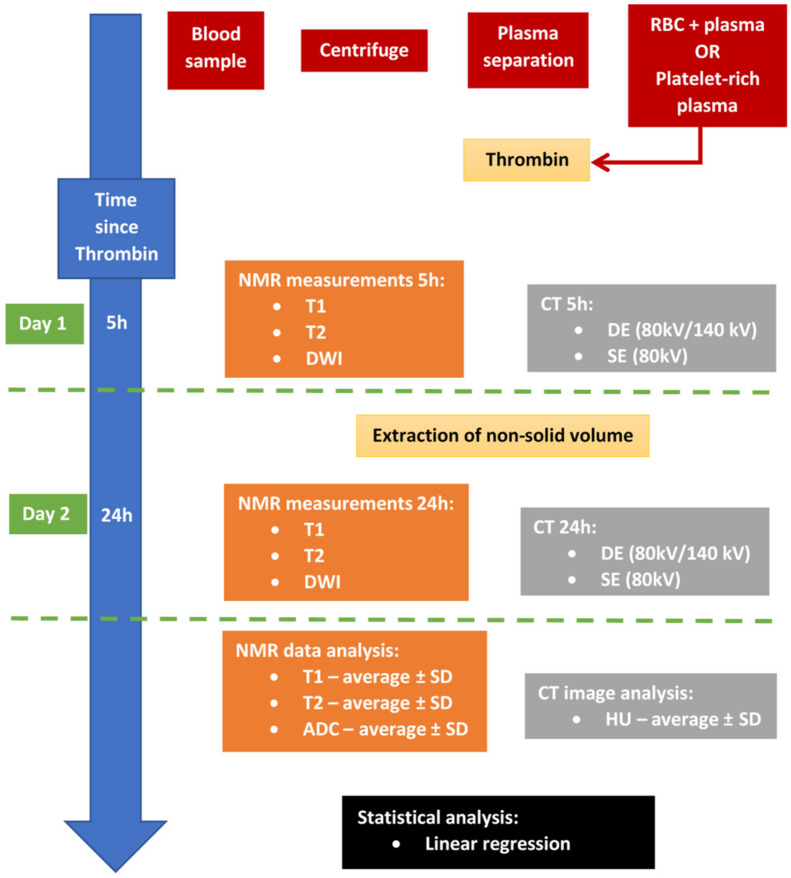
Experiment workflow depicts collected blood sample processing, nuclear magnetic resonance (NMR) measurements and computed tomography (CT) imaging, processing of NMR and CT measurements and statistical analysis. Note that the first set of measurements for red blood cell (RBC) and pure plasma thrombus samples were carried out 5 h after the addition of thrombin, while the first set of measurements for pure platelet thrombus samples were carried out 30 min after they were formed to prevent the samples from drying out.

From collected blood samples, seven different thrombus models (sample types) were made first and then examined using MR and CT scanners to measure various nuclear magnetic resonance (NMR) and CT parameters of the thrombi at multiple time points after their formation, that is, after 5 h (3–6 h) and after 24 h (22–26 h), to see the effect of thrombus retraction [18]. This experiment was repeated 6 times. The measured NMR and CT parameters were also statistically analyzed using linear regression to verify the existence of possible correlations among them. For a clearer depiction, Figure 1 shows the experiment workflow for one sample group.

### 2.1. Artificial Thrombi

Two authors of this study alternately donated blood, one for each experiment replica: one male aged 50–60 and one female aged 20–30. Both were healthy and in good physical condition, with no comorbidities or medication therapy. The blood samples were collected from the cubital vein and stored in 4.5 mL vials (Vacutainer, Becton-Dickinson, Franklin Lakes, NJ, USA) containing 0.45 mL of 0.129 mol/L Na-citrate buffer. Six different RBC thrombus models were made with selected HTs (pure plasma thrombus/0%, 20%, 40%, 60%, 80% and 99%, which was labeled as 100%). In addition, pure platelet thrombi were also made, bringing the number of thrombus models to seven.

To make thrombi with different HTs, the collected blood was centrifuged for 10 min at 3000 rpm (Centrifuge 5810R, Eppendorf, Hamburg, Germany). This resulted in the separation of 99% of the RBCs and plasma. The plasma was removed and saved for future use. Thrombus models with the desired HT were created by mixing the correct ratio of 99% RBCs with plasma; e.g., to make thrombi with 20% HT, 20 µL of 99% RBCs and 80 µL of plasma were mixed in 10 mm diameter Teflon vials, to which 10 µL of thrombin 10 IU (Thrombin, Sigma-Aldrich, Burlington, MA, USA) was added to initiate coagulation. This principle was applied to all remaining model thrombi with different HTs. Pure plasma (0% RBC) was created by mixing 100 µL of centrifuged plasma with 10 µL of thrombin 10 IU. The thrombi prepared in this way were allowed to coagulate for 5 h at room temperature before the first measurements were taken. After the first set of measurements, the thrombi were left at room temperature for an additional 19 h until the second set of measurements. Before these measurements, the thrombi were examined visually, and the serum and RBCs extruded from the retraction process or non-coagulated blood was removed from the sample. The weight of the extruded volume was measured and analyzed.

Platelet thrombus models were made by using platelet-rich plasma, which was created via the centrifugation of the collected blood at a lower speed (1000 rpm) for 15 min (Centrifuge 5810R, Eppendorf, Hamburg, Germany). Platelet-rich plasma was separated from RBC content and divided into 1 mL samples in the 10 mm diameter Teflon vials. To initiate coagulation, 50 µL of CaCl_2_ (320 mmol/L) was added to each sample and mixed thoroughly. To make the platelets stick to each other, thrombin 10 IU (Thrombin, Sigma-Aldrich, Burlington, MA, USA) was added to the sample. This was mixed manually until platelet aggregates were visibly seen sticking together and forming a thrombus. These thrombi had a maximum diameter of 2 mm, which is consistent with the platelet-rich thrombi found in clinical cases. These thrombus models were examined with the first measurements within an hour of their formation to prevent their drying. After the first set of measurements, the thrombi were left at room temperature until the second set of measurements 24 h after their formation. Prior to these measurements, the thrombi were examined visually, and the surrounding serum and RBCs extruded by clot retraction were removed from the sample. The weight of the extruded volume was measured.

### 2.2. Nuclear Magnetic Resonance Measurements and Analysis

NMR measurements were performed on two different NMR/MRI systems. The first was based on a 2.35 T (100 MHz proton frequency) horizontal-bore superconducting magnet (Oxford Instruments, Abingdon, UK) and the second on a 9.4 T (400 MHz proton frequency) vertical-bore superconducting magnet (Jastec, Kobe, Japan). Both systems are equipped with microimaging accessories (Bruker, Ettlingen, Germany) and fully digital spectrometers (Tecmag, Houston, TX, USA). Each thrombus sample was measured in both systems. First, the sample was manually inserted into a 10 mm microimaging radiofrequency (RF) probe, and then its NMR relaxation times *T*_1_ and *T*_2_, as well as the apparent diffusion coefficient (ADC), were measured from signals of the entire sample (in a non-imaging fashion). The measured NMR parameters thus corresponded to their sample average. The longitudinal (*T*_1_) relaxation time was measured using the inversion recovery (IR) pulse sequence with 14 different IR times in a range from 50 ms to 10 s logarithmically equidistant [19]. The transversal (*T*_2_) relaxation time was measured using the Carr–Purcell–Meiboom–Gill (CPMG) sequence with the inter-echo times of 6.1 ms (100 MHz system) and 2.1 ms (400 MHz system) [20]. ADC was measured using the pulsed-gradient spin-echo (PGSE) sequence in 11 different *b*-values in ranges from 0 to 826 s/mm^2^ (100 MHz system) and from 0 to 893 s/mm^2^ (400 MHz system) [21].

### 2.3. Computer Tomography Imaging and Analysis

CT measurements were taken on a dual-source CT (DSCT) Siemens Somatom Drive scanner (Siemens, Erlangen, Germany), with the protocol for pulmonary embolism in dual-energy (DE) (80 kV/140 kV Sn) and single-energy (SE) (80 kV) setups. In both cases, automatic exposure control (AEC) was used to regulate the tube current (mA), with a slice thickness of 1 mm, pitch of 0.55 and matrix of 512 × 512.

The acquired CT images were analyzed using the ImageJ program (NIH, Bethesda, MD, USA) to obtain relevant CT data, namely, the average Hounsfield unit (HU) of each thrombus in the image. This was carried out by setting the region of interest (ROI) to match the region of each individual thrombus that was clearly visible due to the contrast between the Teflon vial and the thrombus. The measurements of the HU parameters of the thrombus were performed 5 h and 24 h after the formation of the thrombus, which made it possible to study the influence of time on the HU parameter for thrombi of different HTs.

### 2.4. Statistical Analysis

The parameters measured using NMR (*T*_1_, *T*_2_, ADC) and the parameters measured using CT (CT number) of the RBC thrombus models were statistically analyzed using linear regression to verify the existence of a possible correlation between the measured parameters and HT and the dependence of this correlation on time from the formation of a thrombus. The linear regression analysis was also used to analyze the dependence of the extruded serum and RBC fraction on HT. The linear regression analysis was performed using Excel (Microsoft, Redmond, WA, USA).

## 3. Results

The retracted thrombus models were analyzed for the extruded serum and RBC fractions (RBC models only), measured using NMR for *T*_1_, *T*_2_ and the ADC parameters and using CT for CT numbers, and, finally, the measured values were analyzed statistically using linear regression.

### 3.1. Extruded Serum and RBC Fraction

**Figure 2:**
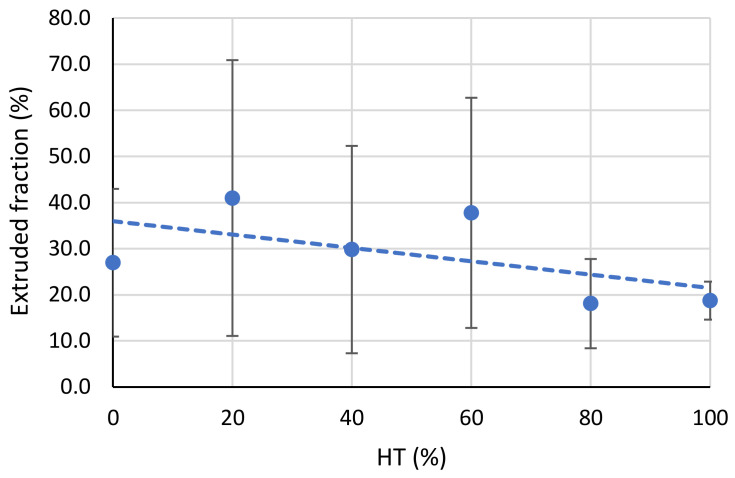
Graph depicts extruded serum and red blood cell (RBC) fraction as a function of hematocrit level (HT), along with a linear regression line showing a weak negative correlation between the two.

The measurements of the extruded serum and RBC fraction, with the linear regression trendline, are presented in Figure 2. The extruded fraction was measured as the ratio between the weight of the extruded volume and the initial weight of the thrombus, where the weight of the extruded volume was obtained as the difference between the initial weight of the thrombus and its weight after blotting the extruded serum and RBCs the next day just before the second round of measurements. The extruded fraction measurements show that the extruded fraction has a slight negative trend with increasing HT; the slope of the linear regression coefficient is −0.14, while its intercept value is 36% (extruded fraction (%) = −0.14 × HT + 36%). However, the corresponding coefficient of determination is low (*R*^2^ = 0.33). Furthermore, no significant differences (*p* > 0.05) were found when comparing the measured fractions of the extruded serum and RBCs between the different HT groups. An analysis of the extruded serum and RBC fractions was performed only for the RBC thrombi, as the platelet thrombi were so small that their extruded volumes were negligible, and their weight could not be reliably measured with the available equipment.

### 3.2. NMR Measurements

Table 1 shows the NMR measurements of the longitudinal (*T*_1_) and transversal (*T*_2_) relaxation times and measurements of the apparent diffusion coefficient (ADC) for the thrombus models measured 5 h and 24 h after their formation. These measurements were made using 100 MHz and 400 MHz NMR systems. All presented data were obtained as the average of six replicate experiments for each thrombus model. Experimental errors correspond to the standard deviations of the measurements within each thrombus model group. The table elements highlighted in red correspond to the RBC thrombus models that overlap in *T*_1_ or *T*_2_ values with the corresponding platelet thrombus model values.

Comparing the RBC and platelet thrombus measurements, there was an overlap in the average *T*_1_ values at 40% HT and 5 h and at 80% HT and 24 h for the 100 MHz MR system, whereas there was no overlap in the measurements for the 400 MHz MR system. There was no overlap in the ADC values in the measurements on either the 100 MHz or 400 MHz MR system. For the *T*_2_ values, there was some overlap at 5 h for both MR systems and at 24 h for the 100 MHz MR system.

**Figure 3:**
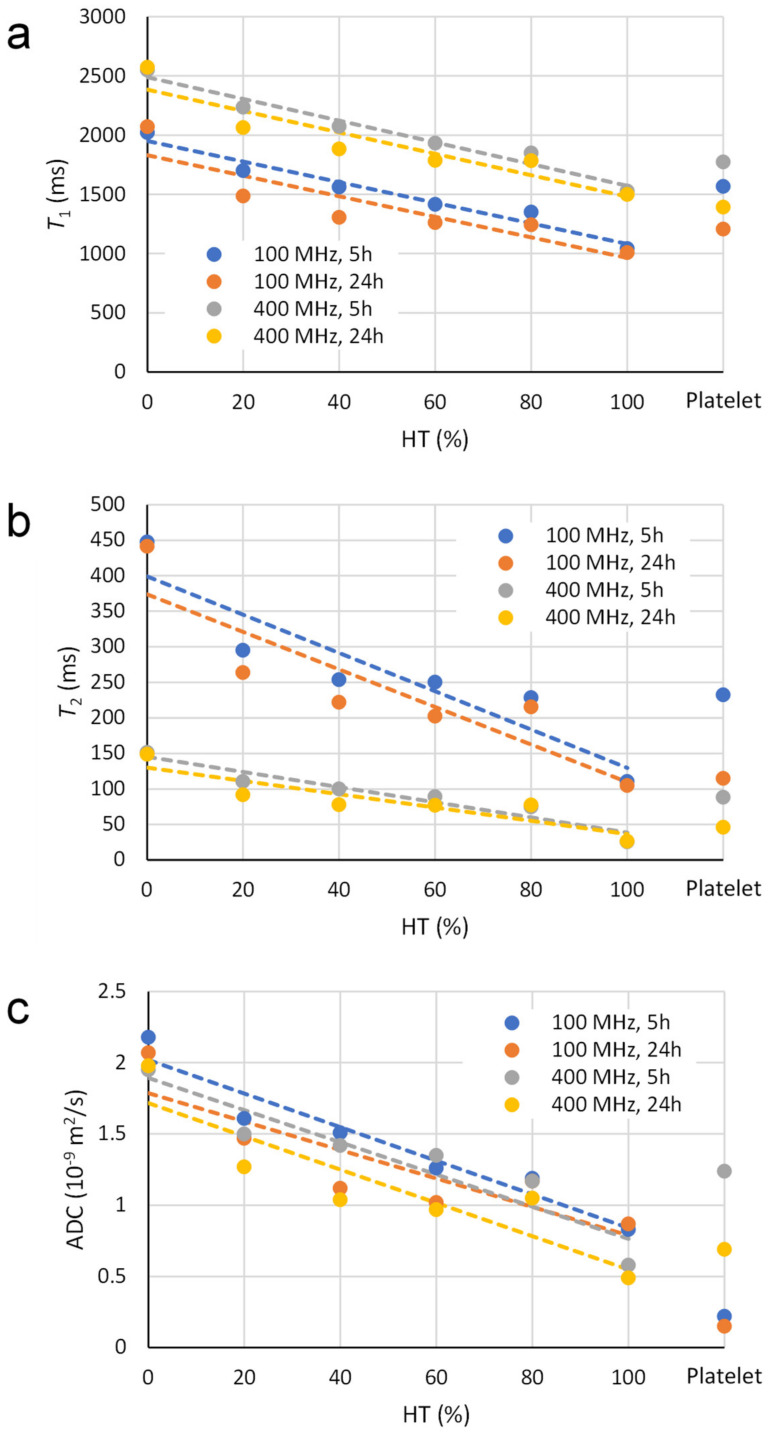
The graphs in panels (**a**–**c**) depict the dependence of measured nuclear magnetic resonance (NMR) *T*_1_, *T*_2_ and apparent diffusion coefficient (ADC) parameters on thrombus type (red blood cell (RBC) vs. platelet) and on hematocrit level (HT) (for RBC thrombi only). Measured values for graphs shown with circles are taken from Table 1, while dashed trendlines are calculated using linear regression analysis. For each of the seven thrombus models (six RBC and one platelet), measurements were carried out for two time points (5 h and 24 h) and with two different NMR systems (100 MHz and 400 MHz).

The measurements in Table 1 are also presented in graphs of *T*_1_, *T*_2_ and ADC as a function of HT, which are shown in Figure 3. From these graphs, it can be seen that all three measured NMR parameters have a negative trend with increasing HT, i.e., negative slopes (*k*) of the trendlines (Table 2). The trends are approximately equally significant for both *T*_2_ (Figure 3b) and ADC (Figure 3c), while they are less significant for *T*_1_ (Figure 3a). This can be confirmed by the parameter 100 × *k*/(50 × *k* + *n*) being significantly higher for ADC and *T*_2_ than for *T*_1_ at both magnetic field strengths (Table 2). As expected, the *T*_1_ values in Figure 3a are longer for measurements at 400 MHz than for measurements at 100 MHz (e.g., 2490 vs. 1950 ms for the trendline *y*-intercept (*n*) at 5 h), while the slopes of the corresponding trendlines are practically identical for both magnetic field strengths (*k* = −8.7 vs. −9.2 ms/% at 5 h). The *T*_2_ measurements in Figure 3b show considerably higher *T*_2_ values at a lower (100 MHz) than at a higher (400 MHz) magnetic field strength (*n* = 400 vs. 145 ms at 5 h); the slopes of the corresponding trendlines are accordingly steeper at 100 MHz than at 400 MHz (*k* = −2.7 vs. −1.1 ms/% at 5 h). In the ADC measurements in Figure 3c, the dependence on the magnetic field strength is almost negligible, while the measurements show lower ADC values in older thrombi (after 24 h) than in fresh thrombi (after 5 h). This difference is approximately 0.2 × 10^−9^ m^2^/s (Table 2). Such a dependence on the age of the thrombus can also be observed in the *T*_1_ and *T*_2_ measurements in Figure 3a,b. According to the linear regression parameters in Table 2, these differences are 120 ms and 110 ms for the *T*_1_ measurements and 25 ms and 15 ms for the *T*_2_ measurements at 100 MHz and 400 MHz, respectively.

### 3.3. CT Measurements

**Figure 4:**
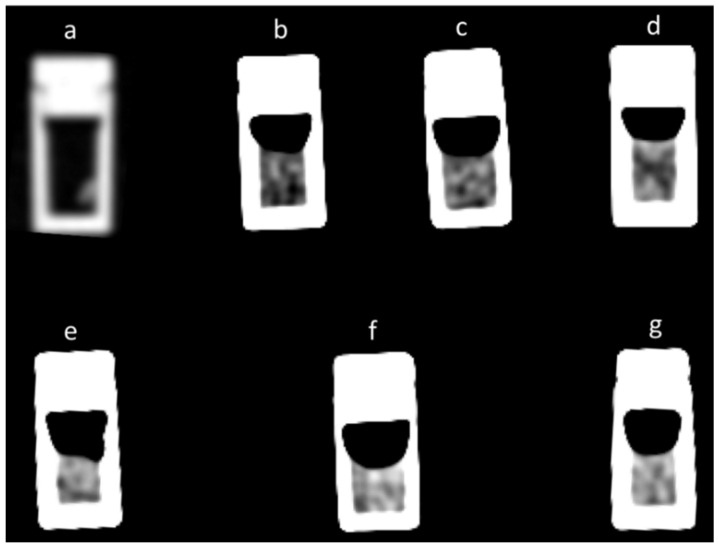
Computed tomography (CT) images in dual-energy (80 kV) mode of all seven different thrombus models taken five hours after initiation of their clotting: platelet thrombus (**a**), pure plasma thrombus (**b**), 20% red blood cell (RBC) thrombus (**c**), 40% RBC thrombus (**d**), 60% RBC thrombus (**e**), 80% RBC thrombus (**f**) and 100% RBC thrombus (**g**). In images (**b**–**g**), presented in the same Window Width (WW) and Window Center (WC), it is clearly seen how the image brightness increases with increasing HT. The platelet thrombus in image (**a**) is displayed with different WW and WC, which is optimal for its presentation.

Figure 4 depicts an example of the CT images of all seven thrombus model samples. The CT numbers of these samples and those of the other five experimental replicates were measured from these and other similar CT images. The results of these measurements are shown in Table 3 with the CT numbers for thrombus modes when measured 5 h and 24 h after their formation, performed in dual-energy (80 kV and 140 kV) and in single-energy (80 kV) CT modes. The CT numbers are presented as the mean and standard deviation of six replicate experiments for each thrombus model.

**Figure 5:**
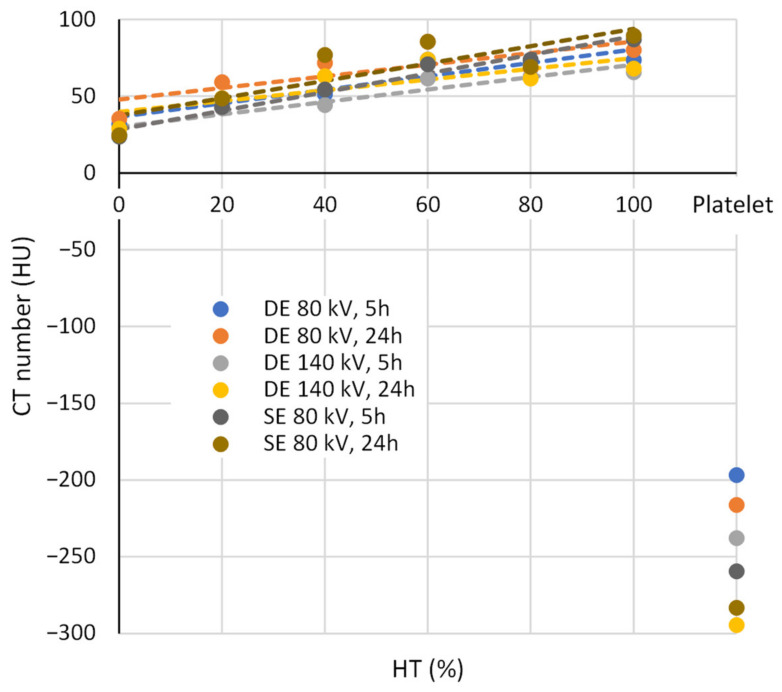
The graph depicts the dependence of computed tomography (CT) numbers on thrombus type (red blood cell (RBC) vs. platelet) and on hematocrit level (HT) (for RBC thrombi only). Measured values for graphs shown with circles are taken from Table 3, while dashed trendlines are calculated using linear regression analysis. For each of seven thrombus models (six RBC and one platelet), measurements were performed for two time points (5 h and 24 h) and with three different CT modes (DE 80 kV, DE 140 kV and SE 80 kV).

The experimental data in Table 3 are also presented in a graph of CT numbers as a function of HU in Figure 5. The experimental points on the graph are presented as circles, while the corresponding dashed trendlines are calculated using a linear regression analysis of the CT data in Table 3 for each of the six different experimental settings: three different CT modes (DE 80 kV, DE 140 kV and SE 80 kV) and two different time points (5 h and 24 h). It can be seen in the graph and in Table 3 that the platelet thrombi have distinctively different CT numbers from the RBC thrombi, so there is no overlap in CT numbers between these two types of thrombi. The CT numbers of the platelet thrombi are negative and, according to the average absolute value, approximately three times higher than the corresponding CT numbers of the RBC thrombi. In addition, the RBC thrombi show an increasing trend of CT numbers with an increasing HU. As can be seen from the linear regression parameters in Table 2, for all CT modes, the fresh thrombi (after 5 h) have higher trendline slopes (k average 0.48 vs. 0.43 HU/%) and lower y-intercepts (*n* average 32 vs. 42 HU) than the older thrombi (after 24 h). Of all the CT modes tested, the slopes are the highest and the y-intercepts are the lowest for the single-energy mode at 80 kV, while for the dual-energy modes, the slopes are lower by approximately 0.2 HU/%, and the y-intercepts are on average 6 HUs higher.

### 3.4. Regression and Statistical Analyses of NMR and CT Measurements

The NMR and CT measurements were analyzed using linear regression. The parameters of this analysis are given in Table 2, where each trendline is characterized by its slope (*k*) and *y*-intercept (*n*), while the goodness of fit between the trendline and the experimental points is given by the coefficient of determination *R*^2^ and by the chi-squared χ^2^. The latter is defined as the weighted sum of squared deviations (the squared difference between the measured and modeled values divided by the variance). The regression parameters *k* and *n* correspond to the trendlines presented in Figure 3 and Figure 5. Table 2 also contains the values of the parameter 100 × *k*/(50 × *k* + *n*), which correspond to the ratio between the difference between the final (100% HT) and the initial (0% HT) values of the trendline and their average. Since this parameter is dimensionless, it can be used to compare the extent of the relative difference of the measured parameter (*T*_1_, *T*_2_, ADC and CT number) over the entire HT range. The parameter with the largest such difference, i.e., with the highest 100 × *k*/(50 × *k* + *n*), can be considered the most sensitive to changes in the RBC thrombus in HT. As can be seen in Table 2, this parameter is the highest for the NMR parameter ADC for the older thrombi and for the fresh thrombi at 400 MHz (highlighted in green), it is relatively high for the NMR parameter *T*_2_ at 400 MHz and for the CT number measured in the SE mode (highlighted in gold), and it is the lowest for the NMR parameter *T*_1_ at 400 MHz (highlighted in red). *R*^2^ values close to one or low χ^2^ values indicate a good fit of the trendline to the measurement. For these measurements, such fits can be considered to be all of those where *R*^2^ > 0.9 or χ^2^ < 10.

## 4. Discussion

This study was designed to investigate the discriminative power of MR and CT in determining the structure and composition of thrombi, i.e., to investigate whether it is possible to distinguish between thrombi composed of RBCs and platelets and to distinguish among thrombi of different HTs based on measurements made by these two methods. This study was conducted on artificial thrombi in vitro; however, its results might also apply to thrombi in vivo provided that such measurements provide equivalent-quality data within a reasonable time.

This study was performed on seven different thrombus models: six RBC models of different HTs and one platelet model. It was found that this number of RBC thrombus models was sufficient to accurately study the dependence of various important image contrast parameters (*T*_1_, *T*_2_, ADC and CT number) on HT using linear regression. Furthermore, the range of HTs from 0% to 100% was higher than that physiologically found in normal blood, i.e., around 40%. The use of a larger HT range can be justified; firstly, it increases the accuracy of the linear regression analysis, and, secondly, contracted thrombi may have HTs that are much higher than normal.

While the results of the serum and RBCs extruded by clot retraction do not show significant differences or strong linear correlations, it is clearly seen that the amount of extruded weight fraction is in general greater in the thrombi with a higher serum content (20%, 40% and 60%). In addition, this result also shows that, for the thrombi with 100% HT, approximately 20% of their volume was extruded after 24 h, although these thrombi contained virtually no serum. Fibrinogen, which is a component of plasma, is essential in hemostasis and thrombosis, as it forms fibrin monomers in the presence of thrombin. These monomers then polymerize and form a fibrin network that gives thrombi their structure [22]. Because thrombi with 100% HT do not have sufficient fibrinogen to form such a network, some blood remains uncoagulated, even after 24 h. The extruded serum mass could not be determined for the platelet thrombi, as their size was too small to obtain enough extruded serum to measure its weight.

The *T*_1_ values show a significant negative linear dependence on HT for both MR systems and for all thrombi. This is due to the increasing content of paramagnetic iron ions in the RBC thrombi and the associated increased effect of *T*_1_ NMR relaxation. Paramagnetic iron was present in hemoglobin in the blood, as we used deoxygenated blood (venous blood), where each hemoglobin group contains four or five unpaired electrons, making hemoglobin paramagnetic. Because of the unpaired electrons, paramagnetic hemoglobin has a magnetic moment, which, due to the thermal motion of the molecules, creates local oscillating fields that shorten the *T*_1_ NMR relaxation time. The shortening of the *T*_1_ relaxation time increases with increasing HTs due to the associated increased concentration of paramagnetic iron [23,24]. In addition to the effect of paramagnetic hemoglobin concertation, there is also an effect of the magnetic field strength on the *T*_1_ NMR relaxation rate; namely, *T*_1_ is longer in a stronger magnetic field than in a weaker one. In our measurements, the difference between the corresponding *T*_1_ relaxation times on the 400 MHz MR system and on the 100 MHz system is in the range of 550 milliseconds (Table 1 and Table 2, and Figure 3a), which is consistent with the literature [25].

As can be seen from the results in Figure 3b, the *T*_2_ measurements have a relatively strong negative linear dependence on HT. This dependency occurs due to an increase in the surface-to-volume ratio with increasing HT, which results in increased surface-induced relaxation and, thus, a decrease in *T*_2_ [26]. This decrease in *T*_2_ occurs because, for a molecule near the surface compared to a molecule in the bulk, the surface molecule is under the influence of paramagnetic centers in the cell or differences in magnetic susceptibility between the cell wall and the plasma-filled extracellular space making the relaxation time faster. Our results show that the *T*_2_ relaxation times measured on the 400 MHz MR system are on average four times shorter than those measured on the 100 MHz MR system (Table 1 and Table 2). This is because the effect of magnetic susceptibility is proportional to the strength of the magnetic field.

Among all measured NMR parameters, ADC had the strongest negative linear dependency on HT in our thrombus models (Figure 3 and Table 2). This dependence can be explained by a decrease in extracellular space and, thus, reduced porosity in such thrombi with increasing HT. Due to the reduced porosity in these thrombi, the diffusion of water molecules in the plasma slows down, and the ADC values decrease. The ADC values were on average lower for the 24 h time point than for the 5 h time point, which may be explained by a further reduction in thrombus porosity due to their retraction at 24 h compared to 5 h. A comparison of the ADC results of our thrombus models between the two NMR systems used showed that the measured ADC values were slightly higher with the 400 MHz system than with the 100 MHz NMR system (Table 1 and Table 2). Since the diffusion does not depend on the magnetic field, there is no physical reason for the difference if the measurements on both NMR systems were performed with the same *b*-values and PGSE time parameters [27]. However, there are still some factors that may contribute to the difference, such as the accuracy of the MR systems, their gradient calibration and the possible temperature difference between the measurements on these two MR systems. Since the measured diffusion was restricted, the difference could also originate from different *b*-values and especially from the temporal parameters of the PGSE sequence. In our case, these parameters were slightly different between the two systems: *b_max_* = 826 s/mm^2^, *Δ* = 20 ms and *δ* = 4 ms compared to *b_max_* = 893 s/mm^2^, *Δ* = 12 ms and *δ* = 2 ms for the 100 MHz and 400 MHz MR systems. The temporal parameter *Δ*, also known as the diffusion time, can have a great influence on the diffusion measurement of a porous system. For a porous system with pore diameter a, the measured diffusion coefficient would be equal to the unrestricted diffusion coefficient $D_{0}$ when *Δ* $\ll a^{2}/6D_{0}$, and it would decrease and level off to $D_{\infty }$ when *Δ* $\gg a^{2}/6D_{0}$, where the ratio $D_{0}/D_{\infty }$ is equal to the tortuosity of the porous medium [28,29]. For *Δ* values between these two extremes, the measured diffusion coefficient is between $D_{\infty }$ and $D_{0}$, and its value depends on the ratio of *Δ* to $a^{2}/6D_{0}$.

The platelet thrombi had values similar to those reported in the literature at 24 h, but they were slightly higher at 5 h. This difference is likely due to the serum and fibrin residues trapped in the platelet thrombi. These were formed when the samples were manually mixed with the addition of thrombin, which stimulates the formation of a fibrin network. The extra serum or plasma trapped in the thrombus was later extruded due to the retraction process, leaving a true platelet thrombus. An overlap of the measured NMR parameter values between the RBC and platelet thrombi occurred in *T*_1_ and *T*_2_ values mostly at HTs above 50%. This result can be explained by the high lipid content of platelets and the fact that the *T*_1_ and *T*_2_ NMR relaxation times of lipid tissues are shorter than those of water-dominated tissues [30]. Negative HU values of platelet thrombi on CT measurements are again probably related to their high lipid content; namely, adipose tissues in humans have a HU between −205 and −51 [31]. While the HU values of our platelet thrombus models are in the negative range, their values are more negative than the minimal adipose tissue value in the literature. This may be due to a partial volume effect in the CT images because the samples were relatively small (2 mm in diameter). This result is promising, as we have demonstrated that CT can clearly differentiate between platelet and RBC thrombi and thrombi with high and low RBC contents. Among the NMR parameters, ADC is without such an overlap of values between RBC and platelet thrombi. In addition, it also has the best sensitivity among the NMR parameters to HT changes in RBC thrombi (parameter 100 × *k*/(50 × *k* + *n*) in Table 2). This result also agrees with our previous findings in a study where ADC and *T*_2_ were used to monitor the progression of thrombolysis [32].

The differences in the linear regression parameters between the NMR measurements on the 100 MHz and 400 MHz NMR systems can be attributed to the higher amount of noise when using the 100 MHz NMR system. When analyzing the CT data, the single-energy CT setting compared to the dual-energy one yielded a greater linear dependence of the average HU on HT, which is a result of greater radiation scattering. However, the dual-energy setting is still a better option when imaging acute ischemic disease. This is because the structure of thrombi is rarely homogenous and may include calcification. These are better imaged in the dual-energy setting, as they have significantly fewer blooming artefacts. A dual-energy setting can provide additional information or a clearer depiction of some structures, with two sets of images being captured [33].

Comparing the linear dependence of the CT and NMR measurements, the results show that the NMR measurements are more precise in measuring RBC content (HT), resulting in a clearer linear dependence and a higher number of significant differences between the different HTs than the CT measurements of the thrombus models with different HTs. This is due to the measured NMR parameters, *T*_1_, *T*_2_ and ADC, which are all sensitive to molecular changes, while CT is only sensitive to the density of imaged tissue. While this is true for in vitro measurements using high-resolution NMR/MRI systems, for in vivo measurements using clinical MRI scanners, current MR technology does not yet provide this precision. Therefore, currently, modern (dual-energy) CT scanners can still serve as a potentially useful tool in the analysis of thrombus structures if the protocol used allows for a sufficient spatial resolution and contrast.

The next step would be to analyze heterogenous thrombi to determine how well different structures can be distinguished within a single thrombus. Although this study was performed on thrombus models, most of the results might also apply to actual thrombi in vivo. Thrombus contraction also occurs in vivo [34], and the measured NMR parameters and CT numbers are the same in thrombus models ex vivo and in actual thrombi in vivo. However, some additional in vivo effects should be considered. In non-occlusive thrombi in vivo, there is also a pulsation of the blood flow, which can cause a slight change in the shape and position of the thrombi. Fast-flowing blood in principle does not produce a detectable MR signal, but this depends on the pulse sequence used. If such an MR signal existed, it could cause motion-type imaging artefacts such that this signal could overlap with the MR signal of the thrombus. However, most of these problems would be reduced with occlusive thrombi. With CT images, there could be a problem with calcifications in the vessel wall; namely, these produce strong CT signals that also extend into their surroundings.

Accurate thrombus characterization is very important for better intervention planning. Imaging techniques such as CT and especially MRI allow for not only the localization of the thrombus and an assessment of its size but also an assessment of its structure and composition, as demonstrated in this study. It has been proven that the outcome of the treatment depends on the structure and composition of the thrombus. This is especially important in the diagnosis and treatment of acute ischemic stroke when time is of the essence. The ability to differentiate between poorly and highly soluble thrombi prior to thrombolytic therapy could help to direct the therapy of poorly soluble thrombi straight to mechanical thrombectomy, which would save time and potentially result in better patient management, better treatment outcomes and less chance of secondary bleeding complications from thrombolytic therapy.

## 5. Conclusions

This study demonstrates that modern dual-energy CT imaging can provide high-quality data that allow for differentiation between thrombi of different compositions. Although NMR relaxometry and diffusometry on high-resolution NMR/MRI systems have been shown to be more efficient and accurate, current clinical MRI scanners cannot achieve this level of performance. With the emerging spectral CT and new and improved MR technologies, it is expected that the current shortcoming of both modalities will be overcome so that more focus can be placed on the characterization of thrombi and their relation to the treatment of ischemic disease.

## Figures and Tables

**Table 1:** Average values and standard deviations of *T*_1_, *T*_2_ and apparent diffusion coefficient (ADC) measurements on both magnetic resonance (MR) systems (100 MHz and 400 MHz) for all thrombus models at both time points (5 h and 24 h). Overlaps in *T*_1_ and *T*_2_ values between platelet thrombi and red blood cell (RBC) thrombi are indicated in red.

		MR System and Measurement Time			
		100 MHz		400 MHz	
	HT (%)	5 h	24 h	5 h	24 h
*T*_1_ (ms)	0	2020 ± 100	2070 ± 80	2550 ± 70	2570 ± 100
	20	1700 ± 180	1490 ± 110	2240 ± 200	2060 ± 80
	40	1560 ± 180	1310 ± 110	2070 ± 140	1880 ± 110
	60	1420 ± 130	1260 ± 190	1930 ± 170	1790 ± 180
	80	1350 ± 80	1250 ± 60	1850 ± 100	1780 ± 70
	100	1040 ± 70	1010 ± 60	1530 ± 110	1500 ± 140
	Platelet	1570 ± 230	1210 ± 150	1780 ± 390	1390 ± 460
*T*_2_ (ms)	0	447 ± 31	442 ± 28	152 ± 8	149 ± 16
	20	295 ± 58	264 ± 47	111 ± 32	92 ± 18
	40	254 ± 42	222 ± 43	100 ± 22	78 ± 18
	60	250 ± 29	202 ± 75	90 ± 15	77 ± 40
	80	229 ± 63	216 ± 67	75 ± 15	78 ± 34
	100	111 ± 13	105 ± 25	26 ± 5	27 ± 9
	Platelet	232 ± 91	115 ± 38	89 ± 24	47 ± 16
ADC (10^−9^ m^2^/s)	0	2.18 ± 0.32	2.07 ± 0.07	1.95 ± 0.04	1.98 ± 0.07
	20	1.61 ± 0.24	1.47 ± 0.18	1.5 ± 0.21	1.27 ± 0.16
	40	1.51 ± 0.21	1.12 ± 0.21	1.42 ± 0.13	1.04 ± 0.2
	60	1.26 ± 0.11	1.02 ± 0.29	1.35 ± 0.11	0.97 ± 0.34
	80	1.19 ± 0.1	1.17 ± 0.25	1.17 ± 0.19	1.05 ± 0.17
	100	0.83 ± 0.13	0.87 ± 0.24	0.58 ± 0.1	0.49 ± 0.11
	Platelet	0.22 ± 0.05	0.15 ± 0.04	1.24 ± 0.37	0.69 ± 0.4

**Table 2:** Linear regression analysis parameters of nuclear magnetic resonance (NMR) and computed tomography (CT) data for red blood cell (RBC) thrombi in Table 1 and Table 3, shown by slopes (*k*) and y-intercept (*n*) values. The goodness of fit between the trendline and the data is given by the coefficient of determination *R*^2^ and the chi-squared χ^2^. In addition, the 100 × *k*/(50 × *k* + *n*) parameter corresponding to the sensitivity of the measured NMR or CT to HT is also shown for each trendline. Cells with high and higher sensitivity parameters are marked in green and gold, and those with low sensitivity are in red.

		NMR						CT		
		*T*_1_		*T*_2_		ADC		DE		SE
		100 MHz	400 MHz	100 MHz	400 MHz	100 MHz	400 MHz	80 kV	140 kV	80 kV
5 h	Slope (*k*)	−8.7 $\frac{\mathrm{ms}}{\%}$	−9.2 $\frac{\mathrm{ms}}{\%}$	−2.7 $\frac{\mathrm{ms}}{\%}$	−1.1 $\frac{\mathrm{ms}}{\%}$	−0.012 $\frac{{\mu m}^{2}}{\mathrm{ms}\%}$	−0.011 $\frac{{\mu m}^{2}}{\mathrm{ms}\%}$	0.44 $\frac{\mathrm{HU}}{\%}$	0.40 $\frac{\mathrm{HU}}{\%}$	0.61 $\frac{\mathrm{HU}}{\%}$
	Intercept (*n*)	1950 ms	2490 ms	400 ms	145 ms	2.0 $\frac{{\mu m}^{2}}{\mathrm{ms}}$	1.9 $\frac{{\mu m}^{2}}{\mathrm{ms}}$	37 HU	30 HU	28 HU
	*R* ^2^	0.96	0.96	0.85	0.92	0.93	0.89	0.86	0.90	0.97
	χ^2^	2.7	2.2	2.3	8.4	6.8	7.5	2.8	1.6	1.6
	100*k*/(50*k* + *n*)	−58%	−45%	−83%	−85%	−101%	−116%	75%	80%	103%
24 h	Slope (*k*)	−8.7 $\frac{\mathrm{ms}}{\%}$	−9.0 $\frac{\mathrm{ms}}{\%}$	−2.6 $\frac{\mathrm{ms}}{\%}$	−0.94 $\frac{\mathrm{ms}}{\%}$	−0.010 $\frac{{\mu m}^{2}}{\mathrm{ms}\%}$	−0.012 $\frac{{\mu m}^{2}}{\mathrm{ms}\%}$	0.38 $\frac{\mathrm{HU}}{\%}$	0.35 $\frac{\mathrm{HU}}{\%}$	0.56 $\frac{\mathrm{HU}}{\%}$
	Intercept (*n*)	1830 ms	2380 ms	375 ms	130 ms	1.8 $\frac{{\mu m}^{2}}{\mathrm{ms}}$	1.7 $\frac{{\mu m}^{2}}{\mathrm{ms}}$	48 HU	40 HU	37 HU
	*R* ^2^	0.80	0.86	0.79	0.79	0.75	0.80	0.62	0.65	0.72
	χ^2^	18.0	11.2	19.4	19.7	9.4	5.2	8.3	7.4	13.1
	100*k*/(50*k* + *n*)	−62%	−47%	−78%	−103%	−109%	−112%	56%	61%	86%

**Table 3:** Average values and standard deviations of computed tomography (CT) numbers in Hounsfield units (HUs) measured in dual energy (DE) (80 kV and 140 kV) and single energy (SE) (80 kV) modes of CT operation for all thrombus models at both time points (5 h and 24 h).

	CT Mode and Measurement Time					
	DE				SE	
	80 kV (HU)		140 kV (HU)		80 kV (HU)	
HT (%)	5 h	24 h	5 h	24 h	5 h	24 h
0	32 ± 10	35 ± 7	25 ± 10	29 ± 7	24 ± 5	25 ± 4
20	48 ± 4	59 ± 9	42 ± 7	49 ± 6	43 ± 7	49 ± 10
40	51 ± 9	72 ± 14	44 ± 9	63 ± 16	54 ± 17	77 ± 21
60	74 ± 14	86 ± 11	61 ± 11	74 ± 9	71 ± 10	85 ± 18
80	73 ± 9	68 ± 13	63 ± 11	61 ± 9	74 ± 15	69 ± 13
100	74 ± 6	80 ± 4	66 ± 6	68 ± 4	87 ± 4	89 ± 7
Platelet	−197 ± 44	−216 ± 117	−238 ± 51	−294 ± 172	−260 ± 111	−283 ± 116

## Data Availability

The data presented in this study are available on request from the corresponding author.
